# Interaction of the New Monofunctional Anticancer Agent Phenanthriplatin With Transporters for Organic Cations

**DOI:** 10.3389/fchem.2018.00180

**Published:** 2018-05-25

**Authors:** Anna Hucke, Ga Young Park, Oliver B. Bauer, Georg Beyer, Christina Köppen, Dorothea Zeeh, Christoph A. Wehe, Michael Sperling, Rita Schröter, Marta Kantauskaitè, Yohannes Hagos, Uwe Karst, Stephen J. Lippard, Giuliano Ciarimboli

**Affiliations:** ^1^Experimental Nephrology, Medical Clinic D, University Hospital, University of Münster, Münster, Germany; ^2^Department of Chemistry, Massachusetts Institute of Technology, Cambridge, MA, United States; ^3^Institute of Inorganic and Analytical Chemistry, University of Münster, Münster, Germany; ^4^European Virtual Institute for Speciation Analysis, Münster, Germany; ^5^PortaCellTec Biosciences GmbH, Göttingen, Germany

**Keywords:** organic cation transporters, platinum compounds, interaction, toxicity, efficacy, side effects

## Abstract

Cancer treatment with platinum compounds is an important achievement of modern chemotherapy. However, despite the beneficial effects, the clinical impact of these agents is hampered by the development of drug resistance as well as dose-limiting side effects. The efficacy but also side effects of platinum complexes can be mediated by uptake through plasma membrane transporters. In the kidneys, plasma membrane transporters are involved in their secretion into the urine. Renal secretion is accomplished by uptake from the blood into the proximal tubules cells, followed by excretion into the urine. The uptake process is mediated mainly by organic cation transporters (OCT), which are expressed in the basolateral domain of the plasma membrane facing the blood. The excretion of platinum into the urine is mediated by exchange with protons via multidrug and toxin extrusion proteins (MATE) expressed in the apical domain of plasma membrane. Recently, the monofunctional, cationic platinum agent phenanthriplatin, which is able to escape common cellular resistance mechanisms, has been synthesized and investigated. In the present study, the interaction of phenanthriplatin with transporters for organic cations has been evaluated. Phenanthriplatin is a high affinity substrate for OCT2, but has a lower apparent affinity for MATEs. The presence of these transporters increased cytotoxicity of phenanthriplatin. Therefore, phenanthriplatin may be especially effective in the treatment of cancers that express OCTs, such as colon cancer cells. However, the interaction of phenanthriplatin with OCTs suggests that its use as chemotherapeutic agent may be complicated by OCT-mediated toxicity. Unlike cisplatin, phenanthriplatin interacts with high specificity with hMATE1 and hMATE2K in addition to hOCT2. This interaction may facilitate its efflux from the cells and thereby decrease overall efficacy and/or toxicity.

## Introduction

Cancer is an important cause of death worldwide (Chakraborty et al., 2018). At present, three major therapeutic strategies to treat cancer are used, namely, surgery, radiotherapy, and chemotherapy (Chakraborty et al., 2018). One of the great advances in the treatment of cancer was the introduction of cisplatin as a chemotherapeutic agent (Rosenberg, 1973). Today, platinum derivatives form part of the chemotherapy of almost every second cancer patient (Galanski et al., 2005; Fennell et al., 2016). The currently approved FDA platinum anticancer agents cisplatin, oxaliplatin, and carboplatin have proved efficacious against a wide variety of tumors, with cisplatin treatment considered to be curative for testicular cancer (Einhorn, 2002; Chovanec et al., 2016). Despite their beneficial effects, treatment with these agents is hampered by the development of drug resistance as well as dose-limiting side effects (Rabik and Dolan, 2007; Kuok et al., 2017). To overcome these problems, alternative metal-based anticancer agents are being developed. Current research efforts focus on a variety of non-classical metal anticancer drug candidates (G Quiroga, 2011; Romero-Canelón and Sadler, 2013; Wang and Gao, 2014; Johnstone et al., 2016; Shahsavani et al., 2016), including non-platinum complexes, Pt(IV) constructs, multinuclear constructs, and monofunctional Pt(II) complexes. Monofunctional agents disobey the classical structure-activity relationships (SARs), which stipulate that active platinum complexes should have two labile leaving groups to form bifunctional adducts with DNA targets in the nucleus (Sherman and Lippard, 1987).

Phenanthriplatin, *cis*-[Pt(NH_3_)_2_Cl(phenanthridine)]^+^, is a monofunctional anticancer agent having greater efficacy and a complimentary spectrum of cancer cell activity compared to that of the clinically approved platinum agents (Park et al., 2012). These differentiating features suggest the possibility for avoiding drug resistance and dose limiting toxicities. Phenanthriplatin forms highly potent monofunctional adducts on DNA (Kellinger et al., 2013) that are capable of inhibiting both RNA and DNA polymerases. Previous research indicated that phenanthriplatin effectively inhibits the ν, ζ, and κ DNA polymerases, as well as the Klenow fragment (Gregory et al., 2014). Upregulation of Pol η, which performs inefficient but high fidelity translesion synthesis (Gregory et al., 2014), has been demonstrated to play a role in the development of resistance to cisplatin (Hicks et al., 2010). The observation that phenanthriplatin is toxic to both Pol η^+^ and Pol η- cells and impervious to other bypass polymerases indicates that phenanthriplatin may escape common cellular resistance mechanisms (Gregory et al., 2014).

In recent years, it has become evident that membrane transporters play an important role not only for resistance to chemotherapeutics, but also for selective uptake of anticancer agents into cancerous cells (Ciarimboli, 2011). Some transporters such as the polyspecific Organic Cation Transporters OCTs (OCT1-3), the Multidrug and Extrusion proteins MATEs (MATE1-2K) and the Copper Transporters 1 and 2 (Ctr1-2) mediate the transport of platinum derivatives through the plasma membrane (Zhang et al., 2006; Lovejoy et al., 2008). Interestingly, OCTs and MATEs share many substrates. Therefore, because of their expression on the basolateral and apical membrane domain, respectively, of secretory epithelial cells, such as those of renal proximal tubules, their concerted action (basolateral uptake by OCTs and apical efflux into the urine by MATEs) mediates the vectorial transport of substrates, resulting in their renal urine excretion (Ciarimboli, 2016). Because transport by MATEs functions as an exchange of substrates with H^+^, the slightly acidic pH in the tubular fluid stimulates secretion processes. Indeed, OCTs and MATEs have been implicated in the development of platinum derivatives side effects such as cisplatin nephrotoxicity (Harrach and Ciarimboli, 2015). It is not yet known whether cellular phenanthriplatin uptake is mediated by membrane transporters. We therefore studied its interaction with OCTs and with the efflux transporters hMATEs.

## Materials and methods

### Cell culture

Experiments were performed with human embryonic kidney (HEK) 293 cells (CRL-1573; American Type Culture Collection, Rochville, MD), which stably express mOCT1, mOCT2, mOCT3 (Schlatter et al., 2014), hOCT1, hOCT2, hOCT3, (kind gift of Prof. H. Koepsell, University Würzburg), hMATE1 or hMATE2K (Schmidt-Lauber et al., 2012). Some experiments were carried out with mouse embryonic fibroblasts (MEFs) from animals with genetic deletion of Ctr1 (Ctr1^−/−^) or not (Ctr1^+/+^) (Nose et al., 2010), provided by Dr. Yasuhiro Nose, Duke University Medical Center, Durham, USA. HEK 293 cells were grown at 37 °C in 50 mL cell culture flasks (Greiner, Frickenhausen, Germany) in DMEM (Biochrom, Berlin, Germany) containing 3.7 g/L NaHCO_3_, 1.0 g/L D-glucose, and 2.0 mM L-glutamine (Biochrom), and gassed with 8 % CO_2_. Penicillin (100 U/mL), 100 mg/L streptomycin (Biochrom), 10 % fetal calf serum, and, only for OCT transfected cells, 0.8 mg/mL geneticin (PAA Laboratories, Coelbe, Germany) were added to the medium. MATE transfected cells were selected with 0.4–0.5 mg/mL hygromycin B (Invitrogen, San Diego, USA). MEFs were cultured in DMEM supplemented with 20% (v/v) heat-inactivated fetal calf serum, 1 × MEM non-essential amino acids (Biochrom), 100 U/mL penicillin/streptomycin, and 55 μM 2-mercaptoethanol (Öhrvik et al., 2013). The medulloblastoma cell line DAOY was obtained from ATCC-LGC (Promochem, Wesel, Germany). The medulloblastoma cell line UW228 cells was obtained from M. Frühwald (University Children's Hospital Münster, Department of Pediatric Hematology and Oncology, Münster, Germany) with kind permission of J. Silber (Department of Neurological Surgery, University of Washington, Seattle, WA). These cells were cultivated in RPMI 1640 medium (Biochrom, Berlin, Germany) supplemented with 1 mM L-glutamine, 100 U/mL penicillin G, 100 μg/mL streptomycin, and 10% fetal calf serum in 25 cm^2^ tissue culture flasks (Greiner Bio One) in a humidified atmosphere of 8% CO_2_ at 37°C.

Experiments were performed with cells grown to confluence for 3–8 days from passages 12–80, depending on the cell type used. Culture and functional analyses of these cells were approved by the state government Landesumweltamt Nordrhein-Westfalen, Essen, Germany (no. 521.-M-1.14/00).

### Fluorescence measurements

The interaction of phenanthriplatin with transporters for organic cations (OCT1-3, MATE1, and MATE2K) was investigated by measuring its effects on the uptake of the fluorescent organic cation 4-(4-dimethylamino)styryl-N-methylpyridinium (ASP^+^). Microfluorometric measurements of ASP^+^ uptake were performed with a fluorescence plate reader (Infinity 200, Tecan, Crailsheim, Germany; excitation at 465 nm, emission at 590 nm) as described previously for cultured HEK 293 cells (Wilde et al., 2009). Before measurements, the culture medium in the wells of the 96 well microtiter plate containing HEK 293 cells was replaced by a ${\text{HCO}}_{3}^{-}$-free Ringer-like solution containing (in mM) NaCl 145, K_2_HPO_4_ 1.6, KH_2_PO_4_ 0.4, D-glucose 5, MgCl_2_ 1, calcium gluconate 1.3, and pH adjusted to 7.4. Fluorescence was measured dynamically at 37°C in each well before and after ASP^+^ addition to ${\text{HCO}}_{3}^{-}$-free Ringer-like solution, alone or together with increasing phenanthriplatin concentrations, and plotted versus time. ASP^+^ (final concentration 1 or 10 μM for experiments with OCTs or MATEs, respectively) was added after the third sampling interval. Emission from the complete area of the well bottom was analyzed four times and averaged for each well. The initial linear fluorescence increase, measured by linear regression within the first 100 s after addition of ASP^+^ alone or together with phenanthriplatin, represents specific cellular uptake of ASP^+^ across plasma membranes and does not reflect possible toxic effects of phenanthriplatin (Ciarimboli et al., 2005a; Wilde et al., 2009). Background fluorescence was measured for each plate in wells with identical solutions containing no cells and subtracted from each well containing cells. Experiments were performed with cells from one passage of the same day with controls and test replicates in the same plate.

### Treatment of cells and preparation of lysates for inductively coupled plasma-mass spectrometry (ICP-MS)

The ICP-MS determinations of cellular platinum content were performed via external calibration using an ICP-MS instrument (iCAP Qc, Thermo Fisher Scientific). Briefly, the culture medium of cells grown to confluence was removed and replaced by fresh complete cell culture medium containing 10 or 100 μM phenanthriplatin. In studies with the hOCT2 transporters, 100 μM cimetidine (Cim) was added to the incubation solution for selected wells. In experiments with hMATE1 and hMATE2K cells, 1 mM 1-methyl-4-phenylpyridinium (MPP^+^) was added to the incubation solution for selected wells as competitor for the transporter-mediated phenanthriplatin cellular accumulation, since it has a similar affinity for hMATE1 and hMATE2K (4.7 and 3.3 μM, respectively, Astorga et al., 2012). After addition of the platinum containing solutions, the cells were incubated for 10 min. Immediately thereafter, the medium was removed and the cells were washed three times with 2 mL of ice cold phosphate-buffered saline (PBS, Biochrom) to remove all extracellular phenanthriplatin. After the PBS was removed, the cells were lysed with 1 mL distilled water. The plates were then incubated for 7.5 min under a microscope at room temperature to observe swelling and bursting of the cells. To ensure complete lysis, this procedure was followed by additional mechanical stress on the cells for 7.5 min on the plate shaker at 400 rpm. The cell lysates were collected with a cell scraper and transferred to 1.5 mL micro-reaction vessels, which were placed in an ultrasonic bath in ice water to destroy residual cell structures. After vortexing and cooled centrifugation (10 min, 4°C, 16,000 g), the supernatant was transferred to polymethylpentene (PMP) vials for storage at −20°C until platinum and protein analyses were performed. An external calibration using a platinum ICP standard (concentration range 10 ng/L to 2 μg/L) was used for quantification. The acquired platinum concentrations were then normalized to the respective protein concentrations obtained via a Bradford assay.

### Bradford assay for protein quantitation

In order to relate Pt quantitation to protein concentration, thereby normalizing for differences in the number of cells, the amount of protein in each sample was analyzed by using the Bradford assay (Bradford, 1976), as previously described (Wehe et al., 2014).

### Cytotoxicity testing

A modified 3-(4,5-dimethylthiazol-2-yl)-2,5-diphenyltetrazolium bromide (MTT) assay (Mosmann, 1983) was used to test the chemosensitivity of cells to phenanthriplatin and, in some experiments, to cisplatin by determining the glycolysis rate via the reduction of the yellow tetrazolium salt MTT to a purple formazan dye, as previously described (Wehe et al., 2014). Briefly, after growing for 24 h in the incubator, cells expressing or not hOCT2, hMATE1, hMATE2 or Ctr1 were treated for 10 min with platinum derivatives. Control cells were incubated with drug-free complete cell culture medium. Afterwards, the medium was carefully removed and replaced by 200 μL of fresh complete cell culture medium. After incubation for another 24 h, 10 μL of MTT solution containing 5 mg/mL of the dye were added to each well, and the cells were again incubated for 3 h. The medium was then removed and 100 μL of lysis buffer containing 10% (w/v) sodium dodecyl sulfate and 40% (v/v) dimethylformamide was added to each well. The plates were shaken for 10 min to destroy the cell structure and dissolve the blue formazan dye (Furchert et al., 2007; Wehe et al., 2014). Finally, the absorbance was measured at 590 nm using an automated microtiter plate reader (Infinite M200; Tecan, Männedorf, Switzerland). The percentage of viable cells in the untreated controls was compared to that for the various treatments. Experiments carried out with DAOY and UW228 cells followed a similar protocol except that these cells were incubated for 24 h with phenanthriplatin.

### Western blotting and SDS-page

For Western blot analysis, DAOY and UW228 cells grown to confluence were solubilized by incubation with PBS containing 1% (w/v) Triton X-100. After 45 min on ice, cell lysates were centrifuged for 1 min at 2,400 g at 4°C. Following this step, the supernatant was diluted in SDS buffer (Roti-Load 1, Roth) with 100 mM dithiothreitol (DTT) and 8% v/v ß-mercaptoethanol and incubated for 5 min at 95°C. The proteins were then separated by SDS-PAGE and transferred to a polyvinylidene fluoride (PVDF) membrane for Western blot analysis. The membranes were blocked with 3% gelatin from cold-water fish skin (Sigma, Munich, Germany). After incubation with the primary antibodies (anti-OCT2 from Alpha Diagnostics, San Antonio, TX, USA at a 1:1,000 dilution and anti-GAPDH from Cell Signaling, Danvers, MA, USA at a 1:2,000 dilution), membranes were incubated with peroxidase-conjugated anti-mouse (DAKO Deutschland, Hamburg, Germany) at a 1:10,000 dilution. Signals were visualized using a Lumi Light detection system (Roche, Mannheim, Germany).

### Statistical analyses

Data are presented as mean values ± SEM, with (n) referring to the number of wells. IC_50_ values defined as the concentration of platinum agent required to inhibit uptake of ASP^+^ by 50% and EC_50_ values used to assess cell viability were obtained by sigmoidal concentration-response curve fitting using GraphPad Prism, Version 5.3 (GraphPad Software, San Diego, USA). Unpaired two-sided Student's *t*-test was employed to prove statistical significance of the effects. For multiple comparisons, ANOVA with Bonferroni post-test was used. A *P*-value < 0.05 was considered statistically significant.

### Solution and chemicals

ASP^+^ was obtained from Molecular Probes (Leiden, The Netherlands). All standard substances were obtained from Sigma (Munich, Germany) or Merck (Darmstadt, Germany) at highest purity available.

## Results

**Figure 1:**
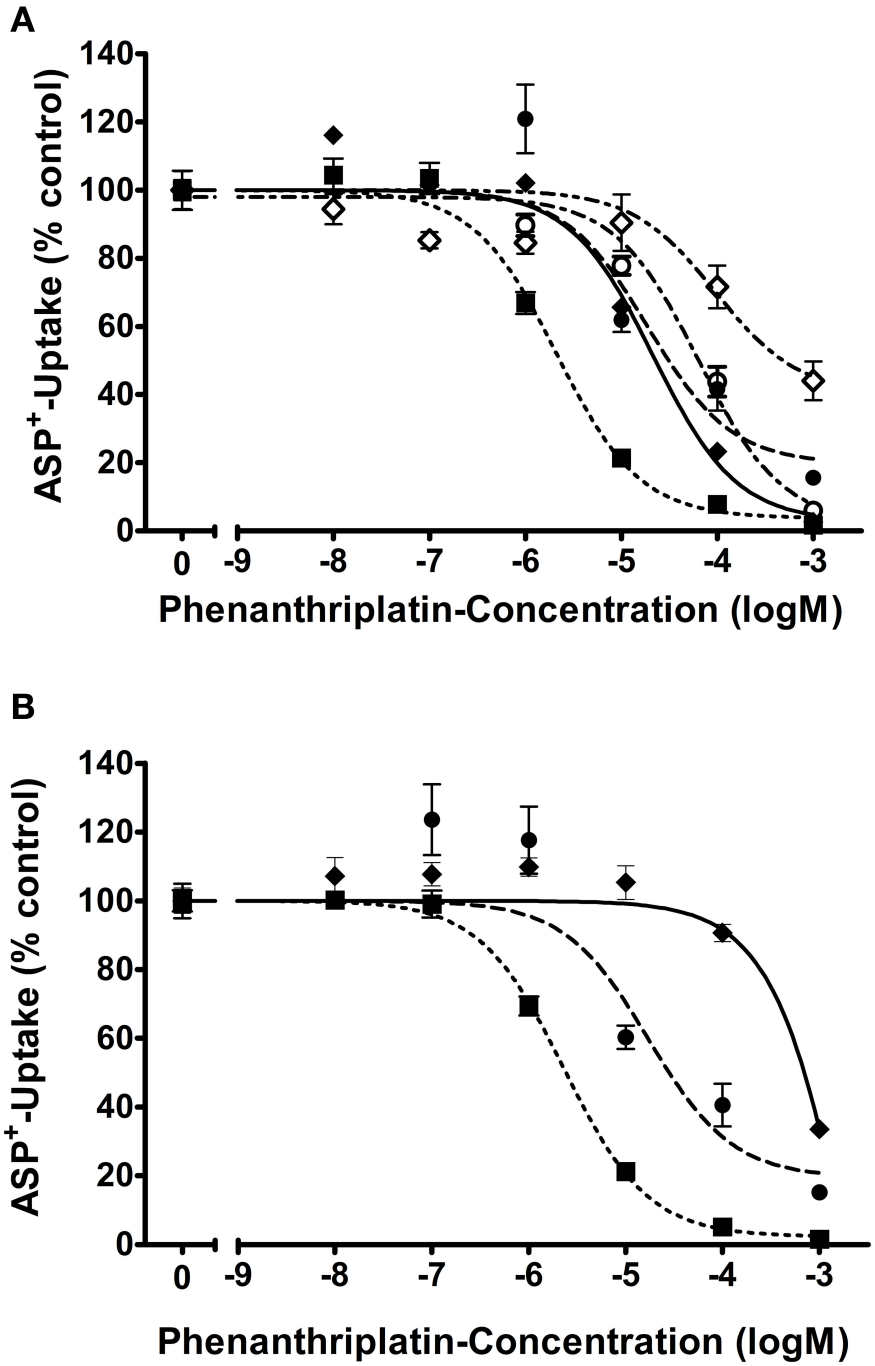
Apparent affinities (IC_50_) of **(A)** hOCT1 (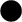; *n* = 6–12), hOCT2 (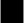; *n* = 6–12), hOCT3 (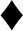; *n* = 6–12), hMATE2K (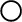; *n* = 15–16), and hMATE1 (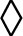; *n* = 7–24) and **(B)** mOCT1 (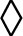; *n* = 6–11), mOCT2 (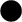; *n* = 6–12), mOCT3 (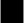; *n* = 6–10) for phenanthriplatin. Values are means ± SEM expressed as % of ASP^+^-uptake in the absence of phenanthriplatin, which was set to 100% (control). The IC_50_ values for the inhibition of initial ASP^+^ uptake by phenanthriplatin were 19, 2, 21, 101, and 57 μM for hOCT1, hOCT2, hOCT3, hMATE1, and hMATE2K, respectively. Those for mOCT1, mOCT2, and mOCT3, were 17, 2, and 3,146 μM, respectively.

First, the uptake of ASP^+^, a known substrate of transporters for organic cations, was investigated in competition with phenanthriplatin. As evident from inspection of Table 1 and Figure 1, similar phenanthriplatin concentrations inhibit ASP^+^ uptake to 50% (IC_50_) for cells overexpressing OCT1 and OCT2 transporters of murine and human origin. The minimum concentration of phenanthriplatin required to inhibit ASP^+^ transport by 50% was 2 μM. This value was determined for cells overexpressing both human or mouse OCT2. In contrast, the source of the OCT3 transporter significantly affects the extent of ASP^+^ inhibition with phenanthriplatin (3,146 μM for mOCT3 vs. 21 μM for hOCT3).

**Table 1:** EC_50_ (μM) values determined for inhibition of ASP^+^ uptake by human and murine OCTs in the presence of phenanthriplatin.

	**Species**	
**OCT subtype**	**Murine**	**Human**
**IC**_50_ **(logEC**_50_ ± **SEM) in** μ**M and number of observations (*****n*****)**		
OCT1	17 (−4.77 ± 0.22)	19 (−4.72 ± 0.21)
	*n* = 6–11	*n* = 6–12
OCT2	2 (−5.64 ± 0.05)	2* (−5.67 ± 0.10)
	*n* = 6–12	*n* = 6–12
OCT3	3,146* (−2.50 ± 1.07)	21^#^ (−4.67 ± 0.07)
	*n* = 6–10	*n* = 6–12
MATE1		101^§^ (−3.99 ± 0.21)
		*n* = 6–12
MATE2K		57^&^ (−4.24 ± 0.07)
		*n* = 15–16

The logIC_50_ values ± SEM and the number of observations are also indicated in parenthesis.

* Statistically significant different from the other paralogs (ANOVA);

§ Statistically significant different from the other paralogs except MATE2K (ANOVA).

& Statistically significant different from the other paralogs except OCT1 and MATE1 (ANOVA).

# *The hashtag (^#^) indicates a statistically significant difference from the murine ortholog (t-test)*.

For what concerns phenanthriplatin interaction with the MATE efflux transporters, a concentration of 101 or 57 μM phenanthriplatin was required to inhibit ASP^+^ transport by 50% in cells overexpressing hMATE1 or hMATE2-K, respectively (Figure 1).

**Figure 2:**
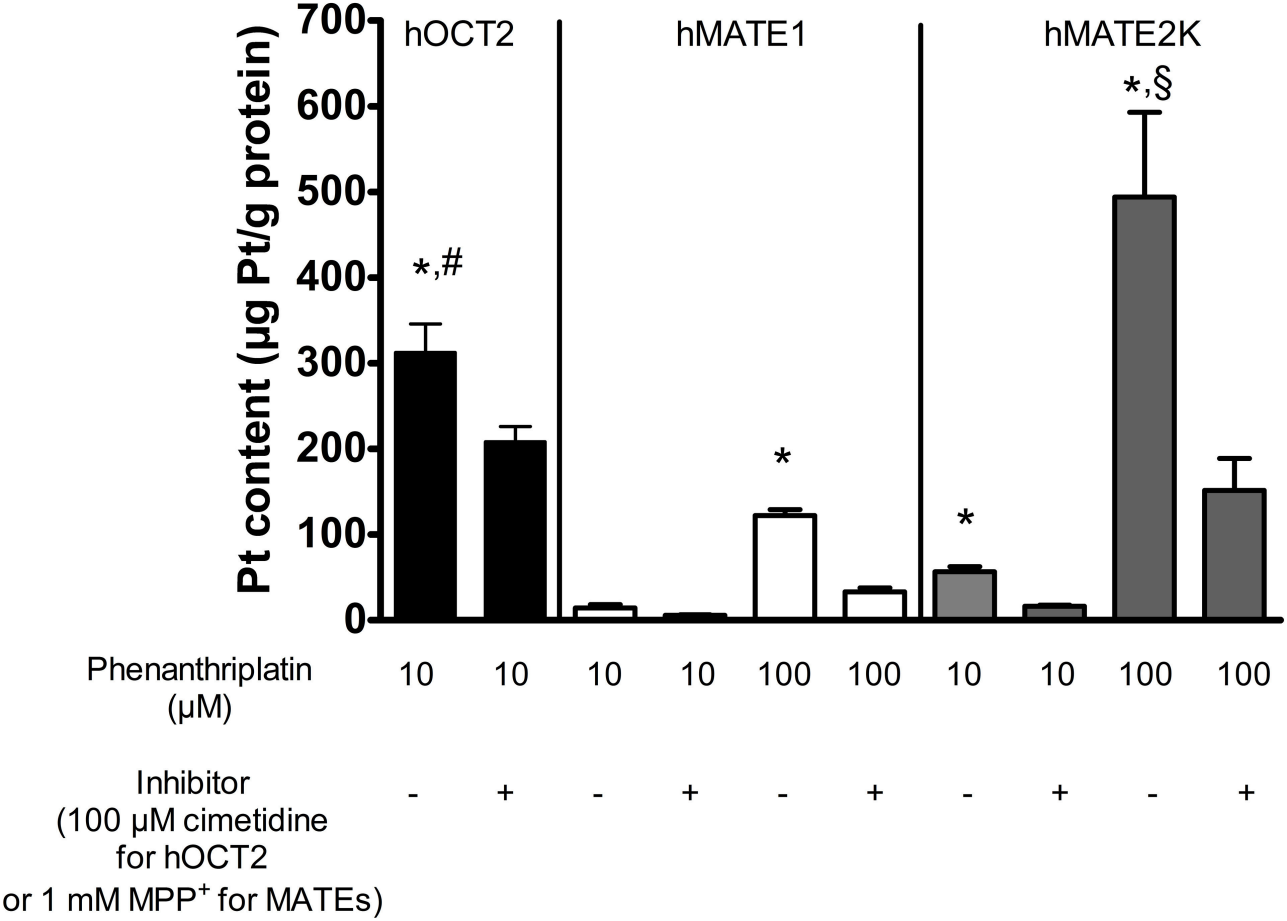
Platinum concentration in lysates from HEK293 cells stably transfected with hOCT2 (black columns), hMATE1 (white columns), or hMATE2K (gray columns) after incubation with phenanthriplatin measured by ICP-MS (*n* = 3–8). The cells were incubated 10 min with 10 μM (hOCT2) or 100 μM (hMATE1 and hMATE2K) phenanthriplatin (Phen) alone or together with 100 μM cimetidine (hOCT2) or 1 mM MPP^+^ (hMATE1 and hMATE2K). ^*^indicates a significant difference from all the respective experiments with inhibitor (unpaired *t*-test); ^#^indicates a significant difference from the other experiments with 10 μM phenanthriplatin alone (ANOVA); ^§^indicates a significant difference from all other experiments in the presence of 100 μM phenanthriplatin (ANOVA).

The cellular accumulation of platinum in HEK 293 cells expressing hOCT2, hMATE1, or hMATE2K was also investigated (Figure 2). Platinum concentrations were determined by ICP-MS following 10 min incubation with 10 or 100 μM phenanthriplatin. When incubated with 10 μM phenanthriplatin, HEK293 cells overexpressing hOCT2 showed significantly higher phenanthriplatin uptake than HEK293 cells expressing hMATE1 or hMATE2K [340 ± 36 (*n* = 8), 15 ± 4 (*n* = 3), and 57 ± 11 (*n* = 3) μg Pt/g protein, respectively, Figure 2]. When phenanthriplatin was co-incubated with 100 μM cimetidine a significant decreased cellular accumulation of platinum in hOCT2 cells was observed (227 ± 19 μg Pt/g protein, *n* = 8). Incubation of hMATE1 and hMATE2K cells with 100 μM phenanthriplatin resulted in a higher cellular phenanthriplatin accumulation than in experiments using 10 μM phenanthriplatin (122 ± 7 and 494 ± 99 μg Pt/g protein, for hMATE1 and hMATE2K, respectively, both *n* = 3, Figure 2), which was significantly inhibited under co-incubation with 1 mM MPP^+^ (33 ± 4 and 151 ± 38 μg Pt/g protein, respectively, both *n* = 3, Figure 2).

These experiments show that both hOCT2 and hMATEs are able to translocate phenanthriplatin across the plasma membrane.

**Figure 3:**
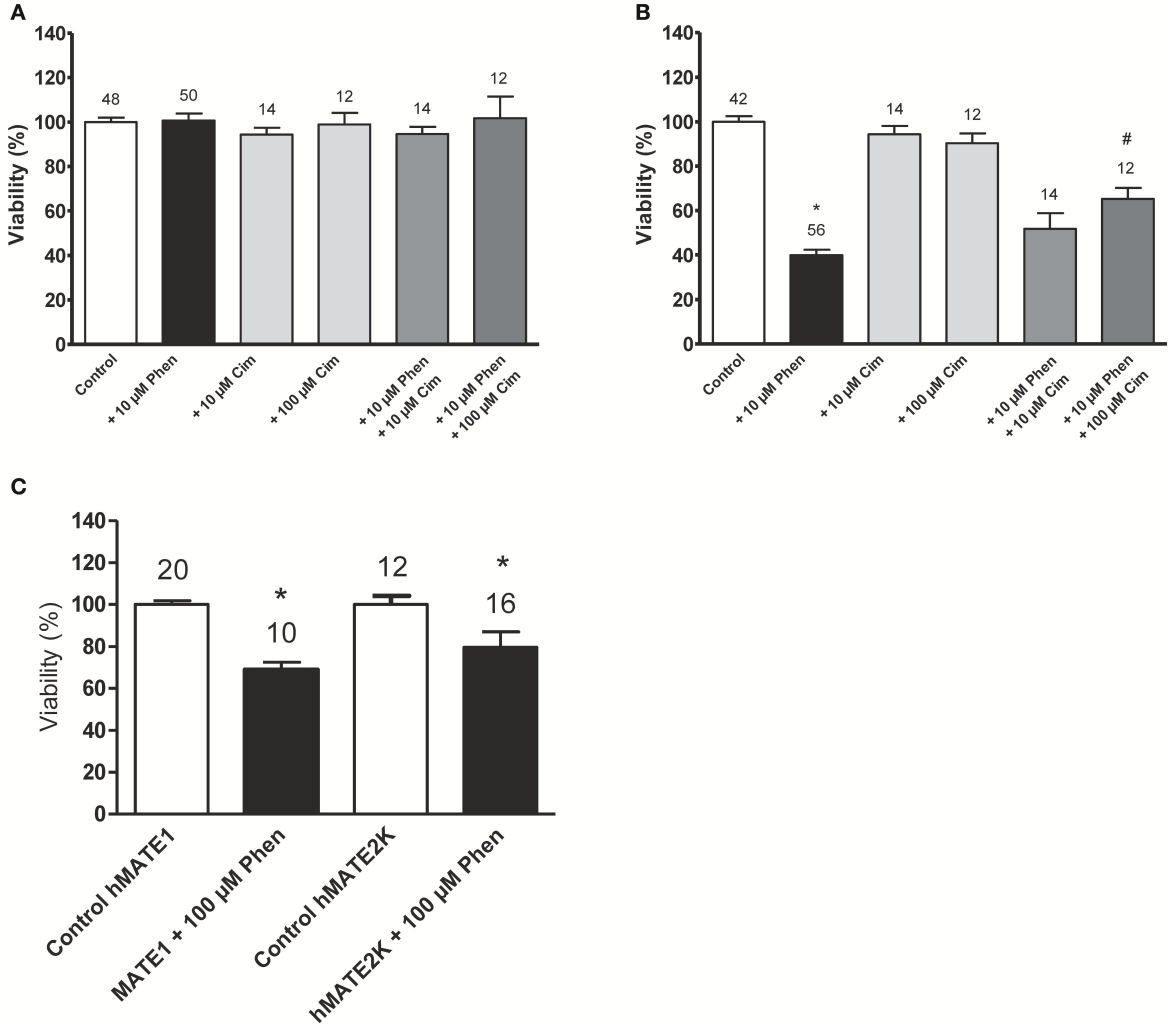
Cell viability (EC_50_) determined by MTT assay for cells incubated with buffer (control); 10 μM phenanthriplatin (Phen); 10 μM cimetidine (Cim); 100 μM cimetidine; 10 μM phenanthriplatin + 10 μM cimetidine; and 10 μM phenanthriplatin + 100 μM cimetidine, for 10 min. **(A)** HEK 293 WT cells **(B)** HEK 293 hOCT2 cells **(C)** HEK293 hMATE1 and hMATE2K cells. Above the columns is the number of experiments. The asterisk (^*^) indicates a statistically significant difference compared to the other columns, ^#^a statistically difference compared to all the other columns except than to 10 μM phenanthriplatin + 10 μM cimetidine (ANOVA).

As shown in Figure 3, the expression of transporters for organic cations is critical for the cellular toxicity of phenanthriplatin. Cell viability studies show that hOCT2 expression in HEK 293 cells is linked to significant phenanthriplatin toxicity which is reduced upon co-incubation of phenanthriplatin with cimetidine (100 μM), a known substrate for hOCT2 (Figure 3B). Cimetidine alone had no significant effect on cell viability. Incubation of cells overexpressing hMATE1 or hMATE2K with 100 μM phenanthriplatin induces significant cell toxicity (Figure 3C).

**Figure 4:**
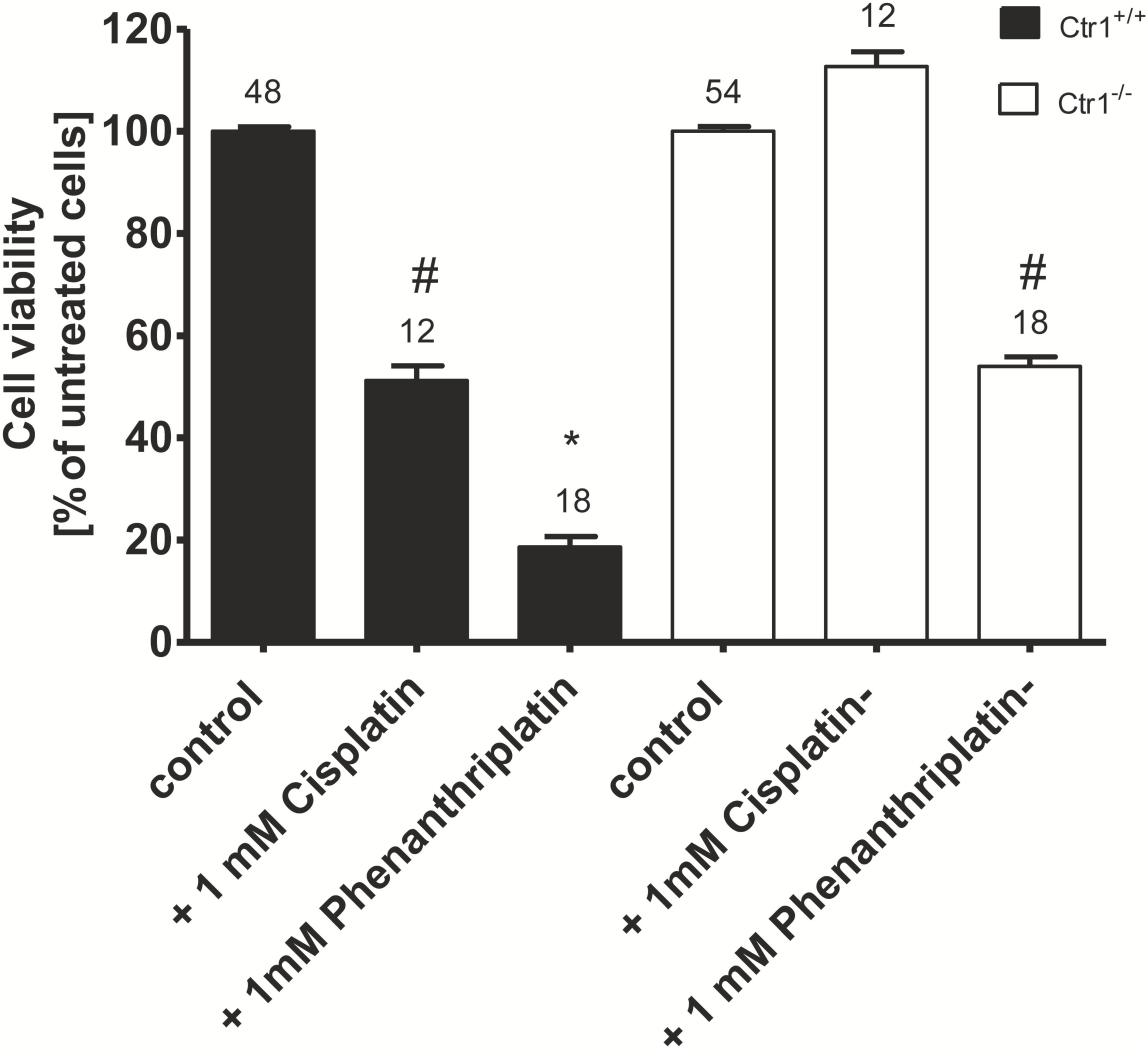
Cell viability of Ctr1^+/+^ and Ctr1^−/−^ MEFs incubated with buffer (control); 1 mM phenanthriplatin or 1 mM cisplatin for 10 min. The number of experiments is listed above each column. The asterisk (^*^) indicates a statistically significant difference compared to the other columns, ^#^a statistically difference compared to all the other columns except than to 1 mM cisplatin in Ctr1^+/+^ MEFs or to 1 mM phenanthriplatin in Ctr1^−/−^ MEFs (ANOVA).

Because the copper transporter 1 (Ctr1) has also been implicated in the cellular uptake of platinum drugs (Holzer et al., 2006), we investigated its possible involvement in phenanthriplatin cellular uptake using MEFs derived from WT (Ctr1^+/+^) and Ctr1^−/−^ mice. We first evaluated whether phenanthriplatin has a toxic effect on MEFs and whether such an effect depends on the expression of Ctr1. Figure 4 shows that phenanthriplatin is toxic both to Ctr1^+/+^ and Ctr1^−/−^ MEFs. Conversely, cisplatin, a known substrate for Ctr1, (Ishida et al., 2002; Holzer et al., 2004), used as a control, was toxic to Ctr1^+/+^ but not to Ctr1^−/−^ MEFs. Lower phenanthriplatin concentrations (<1 mM) did not change the cell viability in Ctr1^+/+^ and Ctr1^−/−^ MEFs (not shown).

**Figure 5:**
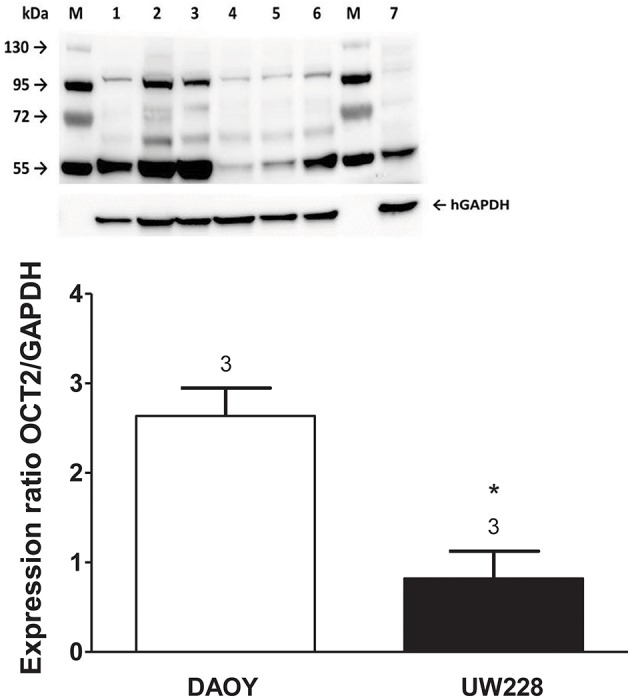
**(Upper)** Western blot analysis of hOCT2 expression in DAOY and UW228 cells. M is the marker lane. 1, 2, 3 are lysates from three different DAOY passages; 4, 5, and 6 are lysates from three different UW228 passages. Lane 7 contains lysate from hOCT2-HEK cells as a control. The respective bands corresponding to the signal of GAPDH are also shown. **(Lower)** Densitometric analysis of the Western blot, showing the hOCT2 expression in relation to the GAPDH expression. Above the columns is the number of experiments. The asterisk (^*^) indicates a statistically significant difference compared to the other column (unpaired *t*-test).

Screening a panel of several tumor cell lines resulted in the identification of two medulloblastoma cell lines, DAOY and UW228, which differed with respect to the expression of mRNA for hOCT2 (Supplementary Figure 2). The differential protein expression of hOCT2 in these two cell lines was determined by Western blot, with DAOY expressing more hOCT2 than UW228 (Figure 5). Also other transporters, such as hMATE1, seem to be expressed at lower level in UW228 than DAOY cells (Supplementary Figure 2).

**Figure 6:**
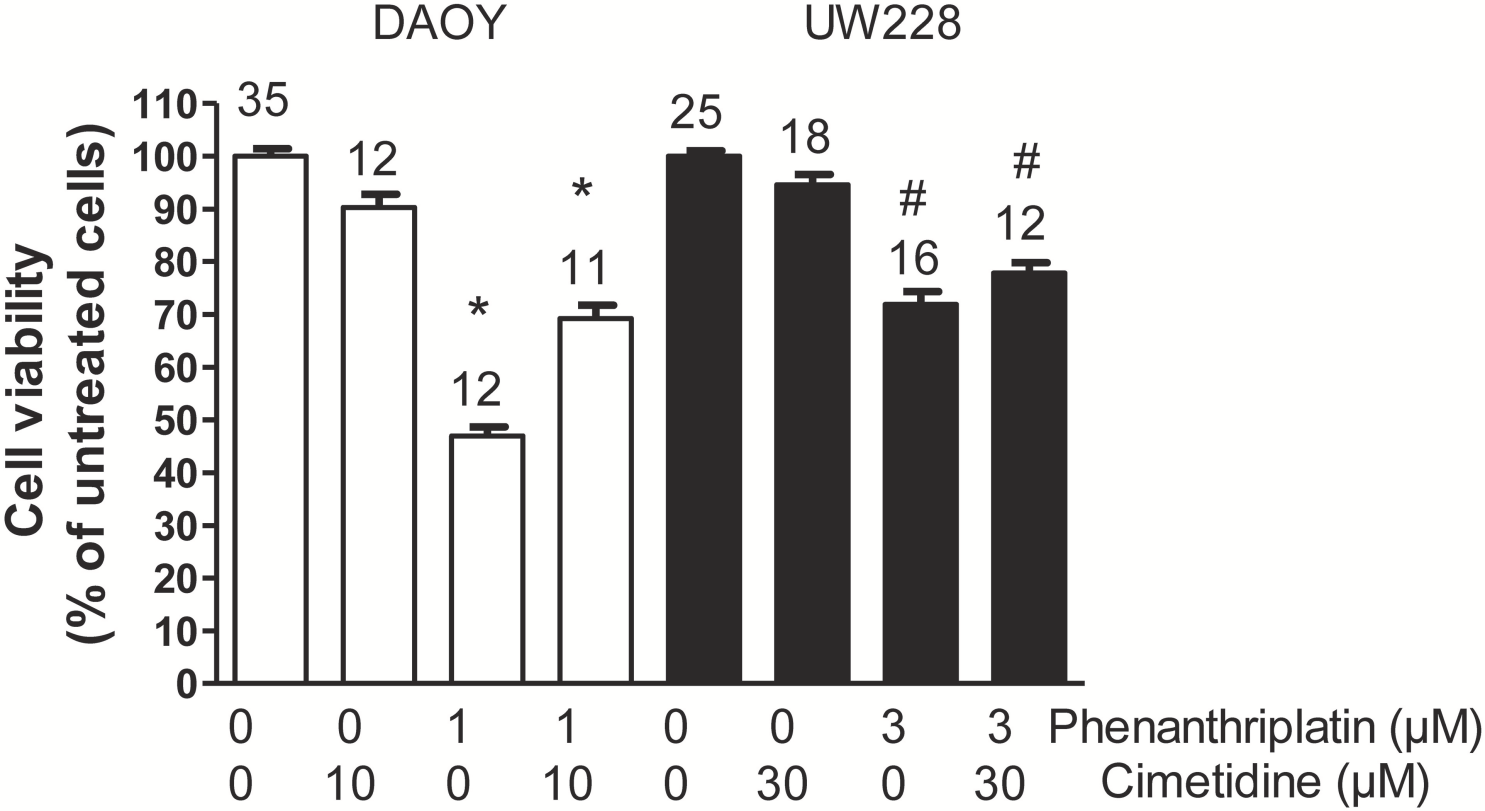
Effects of 24 h incubation of DAOY (empty columns) or UW228 (filled columns) cells with buffer (control) alone and in the presence of 1 or 3 μM phenanthriplatin alone and together with a 10-fold higher concentration of cimetidine on cell viability measured with an MTT-assay. As a further control, experiments performed only in the presence of cimetidine are also shown. Above the columns is the number of experiments. The asterisk (^*^) indicates a statistically significant difference compared to the other columns, ^#^a statistically difference compared to the control experiments (ANOVA).

The determination of EC_50_ for phenanthriplatin toxicity measured by MTT assay after 24 h incubation with 10^−8^–10^−3^ M phenanthriplatin showed that DAOY cells are more sensitive to phenanthriplatin than UW228 cells, EC_50_ = 0.9 vs. 2.8 μM, respectively (Table 2). Using these EC_50_ concentrations, we tested whether competition for uptake by hOCT2 by a 10-fold higher cimetidine concentration can protect these cells from phenanthriplatin toxicity. Only DAOY cells could be efficaciously protected against phenanthriplatin toxicity by cimetidine (Figure 6).

**Table 2:** EC_50_ (logEC_50_ ± SEM) in μM for viability of DAOY and UW228 cells after 24 h incubation with phenanthriplatin.

**DAOY**	**UW228**
**IC**_50_ **(logEC**_50_ ± **SEM) in** μ**M**	
0.9 (−6.05 ± 0.02)	2.8* (−5.55 ± 0.03)
*n* = 23–24	*n* = 24

*n, number of replicates for tested concentration*.

* The asterisk (*) denotes a statistical difference from DAOY, unpaired t-test.

## Discussion

Transporters for organic cations, when expressed in polarized cells such as hepatocytes and renal proximal tubules cells, mediate the vectorial movement of substances through cells, which under physiological conditions results in the secretion of organic cations into the bile and urine, respectively. hOCT1 and hOCT2 are expressed in the basolateral membrane of hepatocytes and renal proximal tubule cells, respectively, whereas hMATEs are localized on the apical domain of these cells (hMATE2K is only expressed in the kidneys; Harrach and Ciarimboli, 2015). For this reason, hOCT1 and hOCT2 mediate the first step of secretion, that is, the uptake of substrates into the cell, and hMATE1 with hMATE2K are important for the final secretion step that delivers the substrates to the bile and urine. These transporters play an important role in the development of side effects caused by platinum drugs, including cisplatin and oxaliplatin. For example, cisplatin causes significant nephrotoxicity, ototoxicity, and peripheral neurotoxicity, whereas oxaliplatin exerts mainly a toxic effect on peripheral nerves (Rabik and Dolan, 2007; Hucke and Ciarimboli, 2016). Cisplatin is a known substrate of hOCT2 (Ciarimboli et al., 2005b) and hOCT3 (Guttmann et al., 2018), and hOCT2-mediated cisplatin uptake has been implicated in the development of side effects (Ciarimboli et al., 2010; Sprowl et al., 2014; Lanvers-Kaminsky et al., 2015, 2017; Lanvers-Kaminsky and Ciarimboli, 2017). Oxaliplatin is also a known substrate for hOCT2 (Zhang et al., 2006; Yokoo et al., 2007; Yonezawa and Inui, 2011), and uptake by this transporter has been linked to the development of peripheral neurotoxicity (Sprowl et al., 2013). Both cisplatin (see Supplementary Figure 1) and oxaliplatin interact with very low affinity with hMATE1, but only oxaliplatin is a substrate of the kidney-specific hMATE2K and is excreted by this transporter in the urine, explaining the lower nephrotoxicity of oxaliplatin (Yokoo et al., 2007; Estrela et al., 2017).

In the present work, we find that phenanthriplatin is a substrate of hOCT2, hMATE1, and hMATE2K. Co-incubation with specific inhibitors significantly decreased phenanthriplatin accumulation by the transporters. Moreover, expression of these transporters correlated well with phenanthriplatin toxicity.

Phenanthriplatin altered the transport of ASP^+^ mediated by hMATE1 and hMATE2K. Compared with the cisplatin inhibition of hMATE1 and hMATE2K (see Supplementary Figure 1), phenanthriplatin is much more able than cisplatin to robustly inhibit the transport of ASP^+^ mediated by hMATE1 and hMATE2K (IC_50_ = 101 and 57 μM, respectively). This observation suggests a potential protective action of hMATEs against eventual cellular toxicity.

Comparison of the IC_50_ values for inhibition of ASP^+^ transport by human and murine OCTs reveals that OCT1 and OCT2 behave the same in the two species. Conversely, large differences in apparent affinities of hOCT3 and mOCT3 were found. Indeed, different properties for rodent and human orthologues have previously been reported (Schlatter et al., 2014). These results are of great importance for the proper interpretation of translational studies (Schlatter et al., 2014).

The presence of Ctr1 sensitizes MEFs to the toxic effects of cisplatin, as confirmed by experiments with Ctr1^−/−^ MEFs, where no decrease in cell viability after treatment with cisplatin was observed (Figure 4). The toxicity of phenanthriplatin was still present in Ctr1^−/−^ MEFs even though at lower level than in Ctr1^+/+^ MEFs. These results suggest that Ctr1 does not have the same importance for phenanthriplatin uptake as it does for cisplatin. Because tumor cells are more sensitive to phenanthriplatin than to cisplatin (Park et al., 2012), we screened the expression of transporters in several tumor cell lines and found that the medulloblastoma cells DAOY and UW228 have different hOCT2 expression, both at the mRNA (not shown) and protein (Figure 5) levels. These cells also display a differential sensitivity to phenanthriplatin. The DAOY cells, which express more hOCT2 and hMATE1, show the greatest increase in sensitivity (Table 2). Inhibition of phenanthriplatin transport through hOCT2 by cimetidine significantly decreased the toxicity of this compound in DAOY but not in UW228 cells, further supporting a role of hOCT2 in amplifying phenanthriplatin toxicity against tumor cells.

## Conclusions

Here we show that phenanthriplatin interacts with transporters for organic cations and that it is even more effective against tumor cells expressing hOCT2. This observation indicates that phenanthriplatin may be especially effective in the treatment of cancers that express OCTs, such as colon cancer cells. However, the interaction of phenanthriplatin with OCTs suggests that its use as chemotherapeutic agent may be complicated by greater OCT-mediated toxicity. Unlike cisplatin, phenanthriplatin interacts with high specificity with hMATE1 and hMATE2K in addition to hOCT2, which may facilitate its efflux from the cells and thereby decrease overall toxicity.

## Author contributions

AH, GYP, OBB, GB, CK, DZ, CAW, MS, RS, MK, YH, and GC performed experiments. AH, GYP, OBB, MS, UK, SJL, and GC planned the study and wrote the manuscript.

### Conflict of interest statement

The authors declare that the research was conducted in the absence of any commercial or financial relationships that could be construed as a potential conflict of interest.

## References

[B1] Astorga B. Ekins S. Morales M. Wright S. H. (2012). Molecular determinants of ligand selectivity for the human multidrug and toxin extruder proteins MATE1 and MATE2-K. J. Pharmacol. Exp. Ther. 341, 743–755. 10.1124/jpet.112.191577 22419765 PMC3362881

[B2] Bradford M. M. (1976). A rapid and sensitive method for the quantitation of microgram quantities of protein utilizing the principle of protein-dye binding. Anal. Biochem. 72, 248–254. 10.1016/0003-2697(76)90527-3 942051

[B3] Chakraborty C. Sharma A. R. Sharma G. Sarkar B. K. Lee S. S. (2018). The novel strategies for next-generation cancer treatment: miRNA combined with chemotherapeutic agents for the treatment of cancer. Oncotarget 9, 10164–10174. 10.18632/oncotarget.24309 29515800 PMC5839381

[B4] Chovanec M. Hanna N. Cary K. C. Einhorn L. Albany C. (2016). Management of stage I testicular germ cell tumours. Nat. Rev. Urol. 13, 663–673. 10.1038/nrurol.2016.164 27618772

[B5] Ciarimboli G. (2011). Role of organic cation transporters in drug-induced toxicity. Expert. Opin. Drug Metab. Toxicol. 7, 159–174. 10.1517/17425255.2011.547474 21241199

[B6] Ciarimboli G. (2016). Introduction to the cellular transport of organic cations, in Organic Cation Transporters, eds Ciarimboli G. Gautron S. Schlatter E. (Heidelberg; New York, NY; Dordrecht; London: Springer), 1–48.

[B7] Ciarimboli G. Deuster D. Knief A. Sperling M. Holtkamp M. Edemir B.. (2010). Organic cation transporter 2 mediates cisplatin-induced oto- and nephrotoxicity and is a target for protective interventions. Am. J. Pathol. 176, 1169–1180. 10.2353/ajpath.2010.090610 20110413 PMC2832140

[B8] Ciarimboli G. Koepsell H. Iordanova M. Gorboulev V. Dürner B. Lang D.. (2005a). Individual PKC-phosphorylation sites in organic cation transporter 1 determine substrate selectivity and transport regulation. J. Am. Soc. Nephrol. 16, 1562–1570. 10.1681/ASN.2004040256 15829703

[B9] Ciarimboli G. Ludwig T. Lang D. Pavenstädt H. Koepsell H. Piechota H. J.. (2005b). Cisplatin nephrotoxicity is critically mediated via the human organic cation transporter 2. Am. J. Pathol. 167, 1477–1484. 10.1016/S0002-9440(10)61234-5 16314463 PMC1613191

[B10] Einhorn L. H. (2002). Curing metastatic testicular cancer. Proc. Natl. Acad. Sci. U.S.A. 99, 4592–4595. 10.1073/pnas.072067999 11904381 PMC123692

[B11] Estrela G. R. Wasinski F. Felizardo R. J. F. Souza L. L. Câmara N. O. S. Bader M.. (2017). MATE-1 modulation by kinin B1 receptor enhances cisplatin efflux from renal cells. Mol. Cell Biochem. 428, 101–108. 10.1007/s11010-016-2920-x 28161805

[B12] Fennell D. A. Summers Y. Cadranel J. Benepal T. Christoph D. C. Lal R.. (2016). Cisplatin in the modern era: the backbone of first-line chemotherapy for non-small cell lung cancer. Cancer Treat. Rev. 44, 42–50. 10.1016/j.ctrv.2016.01.003 26866673

[B13] Furchert S. E. Lanvers-Kaminsky C. Juürgens H. Jung M. Loidl A. Frühwald M. C. (2007). Inhibitors of histone deacetylases as potential therapeutic tools for high-risk embryonal tumors of the nervous system of childhood. Int. J. Cancer 120, 1787–1794. 10.1002/ijc.22401 17230517

[B14] Galanski M. Jakupec M. A. Keppler B. K. (2005). Update of the preclinical situation of anticancer platinum complexes: novel design strategies and innovative analytical approaches. Curr. Med. Chem. 12, 2075–2094. 10.2174/0929867054637626 16101495

[B15] G Quiroga A. (2011). Non-classical structures among current platinum complexes with potential as antitumor drugs. Curr. Top. Med. Chem. 11, 2613–2622. 10.2174/156802611798040723 22039872

[B16] Gregory M. T. Park G. Y. Johnstone T. C. Lee Y. S. Yang W. Lippard S. J. (2014). Structural and mechanistic studies of polymerase eta bypass of phenanthriplatin DNA damage. Proc. Natl. Acad. Sci. U.S.A. 111, 9133–9138. 10.1073/pnas.1405739111 24927576 PMC4078841

[B17] Guttmann S. Chandhok G. Groba S. R. Niemietz C. Sauer V. Gomes A.. (2018). Organic cation transporter 3 mediates cisplatin and copper cross-resistance in hepatoma cells. Oncotarget 9, 743–754. 10.18632/oncotarget.23142 29416650 PMC5787505

[B18] Harrach S. Ciarimboli G. (2015). Role of transporters in the distribution of platinum-based drugs. Front. Pharmacol. 6:85. 10.3389/fphar.2015.00085 25964760 PMC4408848

[B19] Hicks J. K. Chute C. L. Paulsen M. T. Ragland R. L. Howlett N. G. Guéranger Q.. (2010). Differential roles for DNA polymerases eta, zeta, and REV1 in lesion bypass of intrastrand versus interstrand DNA cross-links. Mol. Cell Biol. 30, 1217–1230. 10.1128/MCB.00993-09 20028736 PMC2820889

[B20] Holzer A. K. Manorek G. H. Howell S. B. (2006). Contribution of the major copper influx transporter CTR1 to the cellular accumulation of cisplatin, carboplatin, and oxaliplatin. Mol. Pharmacol. 70, 1390–1394. 10.1124/mol.106.022624 16847145

[B21] Holzer A. K. Samimi G. Katano K. Naerdemann W. Lin X. Safaei R.. (2004). The copper influx transporter human copper transport protein 1 regulates the uptake of cisplatin in human ovarian carcinoma cells. Mol. Pharmacol. 66, 817–823. 10.1124/mol.104.001198 15229296

[B22] Hucke A. Ciarimboli G. (2016). The role of transporters in the toxicity of chemotherapeutic drugs: focus on transporters for organic cations. J. Clin. Pharmacol. 56, S157–S172. 10.1002/jcph.706 27385173

[B23] Ishida S. Lee J. Thiele D. J. Herskowitz I. (2002). Uptake of the anticancer drug cisplatin mediated by the copper transporter Ctr1 in yeast and mammals. Proc. Natl. Acad. Sci. U.S.A. 99, 14298–14302. 10.1073/pnas.162491399 12370430 PMC137878

[B24] Johnstone T. C. Suntharalingam K. Lippard S. J. (2016). The next generation of platinum drugs: targeted Pt(II) agents, nanoparticle delivery, and Pt(IV) prodrugs. Chem. Rev. 116, 3436–3486. 10.1021/acs.chemrev.5b00597 26865551 PMC4792284

[B25] Kellinger M. W. Park G. Y. Chong J. Lippard S. J. Wang D. (2013). Effect of a monofunctional phenanthriplatin-DNA adduct on RNA polymerase II transcriptional fidelity and translesion synthesis. J. Am. Chem. Soc. 135, 13054–13061. 10.1021/ja405475y 23927577 PMC3791135

[B26] Kuok K. I. Li S. Wyman I. W. Wang R. (2017). Cucurbit[7]uril: an emerging candidate for pharmaceutical excipients. Ann. N.Y. Acad. Sci. 1398, 108–119. 10.1111/nyas.13376 28692768

[B27] Lanvers-Kaminsky C. Ciarimboli G. (2017). Pharmacogenetics of drug-induced ototoxicity caused by aminoglycosides and cisplatin. Pharmacogenomics 18, 1683–1695. 10.2217/pgs-2017-0125 29173064

[B28] Lanvers-Kaminsky C. Sprowl J. A. Malath I. Deuster D. Eveslage M. Schlatter E.. (2015). Human OCT2 variant c.808G>T confers protection effect against cisplatin-induced ototoxicity. Pharmacogenomics 16, 323–332. 10.2217/pgs.14.182 25823781 PMC4865798

[B29] Lanvers-Kaminsky C. Zehnhoff-Dinnesen A. A. Parfitt R. Ciarimboli G. (2017). Drug-induced ototoxicity: mechanisms, pharmacogenetics, and protective strategies. Clin. Pharmacol. Ther. 101, 491–500. 10.1002/cpt.603 28002638

[B30] Lovejoy K. S. Todd R. C. Zhang S. McCormick M. S. D'Aquino J. A. Reardon J. T.. (2008). cis-Diammine(pyridine)chloroplatinum(II), a monofunctional platinum(II) antitumor agent: uptake, structure, function, and prospects. Proc. Natl. Acad. Sci. U.S.A. 105, 8902–8907. 10.1073/pnas.0803441105 18579768 PMC2449337

[B31] Mosmann T. (1983). Rapid colorimetric assay for cellular growth and survival: application to proliferation and cytotoxicity assays. J. Immunol. Methods 65, 55–63. 10.1016/0022-1759(83)90303-4 6606682

[B32] Nose Y. Wood L. K. Kim B. E. Prohaska J. R. Fry R. S. Spears J. W.. (2010). Ctr1 is an apical copper transporter in mammalian intestinal epithelial cells *in vivo* that is controlled at the level of protein stability. J. Biol. Chem. 285, 32385–32392. 10.1074/jbc.M110.143826 20699218 PMC2952240

[B33] Öhrvik H. Nose Y. Wood L. K. Kim B. E. Gleber S. C. Ralle M.. (2013). Ctr2 regulates biogenesis of a cleaved form of mammalian Ctr1 metal transporter lacking the copper- and cisplatin-binding ecto-domain. Proc. Natl. Acad. Sci. U.S.A. 110, E4279–E4288. 10.1073/pnas.1311749110 24167251 PMC3831961

[B34] Park G. Y. Wilson J. J. Song Y. Lippard S. J. (2012). Phenanthriplatin, a monofunctional DNA-binding platinum anticancer drug candidate with unusual potency and cellular activity profile. Proc. Natl. Acad. Sci. U.S.A. 109, 11987–11992. 10.1073/pnas0.1207670109 22773807 PMC3409760

[B35] Rabik C. A. Dolan M. E. (2007). Molecular mechanisms of resistance and toxicity associated with platinating agents. Cancer Treat. Rev. 33, 9–23. 10.1016/j.ctrv.2006.09.006 17084534 PMC1855222

[B36] Romero-Canelón I. Sadler P. J. (2013). Next-generation metal anticancer complexes: multitargeting via redox modulation. Inorg. Chem. 52, 12276–12291. 10.1021/ic400835n 23879584

[B37] Rosenberg B. (1973). Platinum coordination complexes in cancer chemotherapy. Naturwissenschaften 60, 399–406. 10.1007/BF00623551 4203933

[B38] Schlatter E. Klassen P. Massmann V. Holle S. K. Guckel D. Edemir B.. (2014). Mouse organic cation transporter 1 determines properties and regulation of basolateral organic cation transport in renal proximal tubules. Pflug. Arch. 466, 1581–1589. 10.1007/s00424-013-1395-9 24233562

[B39] Schmidt-Lauber C. Harrach S. Pap T. Fischer M. Victor M. Heitzmann M.. (2012). Transport mechanisms and their pathology-induced regulation govern tyrosine kinase inhibitor delivery in rheumatoid arthritis. PLoS ONE 7:e52247. 10.1371/journal.pone.0052247 23284953 PMC3527388

[B40] Shahsavani M. B. Ahmadi S. Aseman M. D. Nabavizadeh S. M. Rashidi M. Asadi Z.. (2016). Anticancer activity assessment of two novel binuclear platinum (II) complexes. J. Photochem. Photobiol. B 161, 345–354. 10.1016/j.jphotobiol.2016.05.025 27289447

[B41] Sherman S. E. Lippard S. J. (1987). Structural aspects of platinum anticancer drug-interactions with Dna. Chem. Rev. 87, 1153–1181 10.1021/cr00081a013

[B42] Sprowl J. A. Ciarimboli G. Lancaster C. S. Giovinazzo H. Gibson A. A. Du G.. (2013). Oxaliplatin-induced neurotoxicity is dependent on the organic cation transporter OCT2. Proc. Natl. Acad. Sci. U.S.A. 110, 11199–11204. 10.1073/pnas.1305321110 23776246 PMC3704038

[B43] Sprowl J. A. Lancaster C. S. Pabla N. Hermann E. Kosloske A. M. Gibson A. A.. (2014). Cisplatin-induced renal injury is independently mediated by OCT2 and p53. Clin. Cancer Res. 20, 4026–4035. 10.1158/1078-0432.CCR-14-0319 24916697 PMC4119572

[B44] Wang K. Gao E. (2014). Recent advances in multinuclear complexes as potential anticancer and DNA binding agents. Anticancer Agents Med. Chem. 14, 147–169. 10.2174/18715206113139990313 23869783

[B45] Wehe C. A. Beyer G. Sperling M. Ciarimboli G. Karst U. (2014). Assessing the intracellular concentration of platinum in medulloblastoma cell lines after Cisplatin incubation. J. Trace Elem. Med. Biol. 28, 166–172. 10.1016/j.jtemb.2014.01.001 24560561

[B46] Wilde S. Schlatter E. Koepsell H. Edemir B. Reuter S. Pavenstädt H.. (2009). Calmodulin-associated post-translational regulation of rat organic cation transporter 2 in the kidney is gender dependent. Cell Mol. Life Sci. 66, 1729–1740. 10.1007/s00018-009-9145-z 19330287 PMC11115569

[B47] Yokoo S. Yonezawa A. Masuda S. Fukatsu A. Katsura T. Inui K. (2007). Differential contribution of organic cation transporters, OCT2 and MATE1, in platinum agent-induced nephrotoxicity. Biochem. Pharmacol. 74, 477–487 10.1016/j.bcp.2007.03.004 17582384

[B48] Yonezawa A. Inui K. (2011). Organic cation transporter OCT/SLC22A and H(+)/organic cation antiporter MATE/SLC47A are key molecules for nephrotoxicity of platinum agents. Biochem. Pharmacol. 81, 563–568. 10.1016/j.bcp.2010.11.016 21144842

[B49] Zhang S. Lovejoy K. S. Shima J. E. Lagpacan L. L. Shu Y. Lapuk A.. (2006). Organic cation transporters are determinants of oxaliplatin cytotoxicity. Cancer Res. 66, 8847–8857 10.1158/0008-5472.CAN-06-0769 16951202 PMC2775093

