# Supplementary material for: Interaction of the New Monofunctional Anticancer Agent Phenanthriplatin With Transporters for Organic Cations
Source: Front Chem. 2018 May 25;6:180. doi: 10.3389/fchem.2018.00180 (PMC5982655; doi:10.3389/fchem.2018.00180)
Supplement: Supplementary file 1 [file Image_1.PDF]

Supplementary Materials

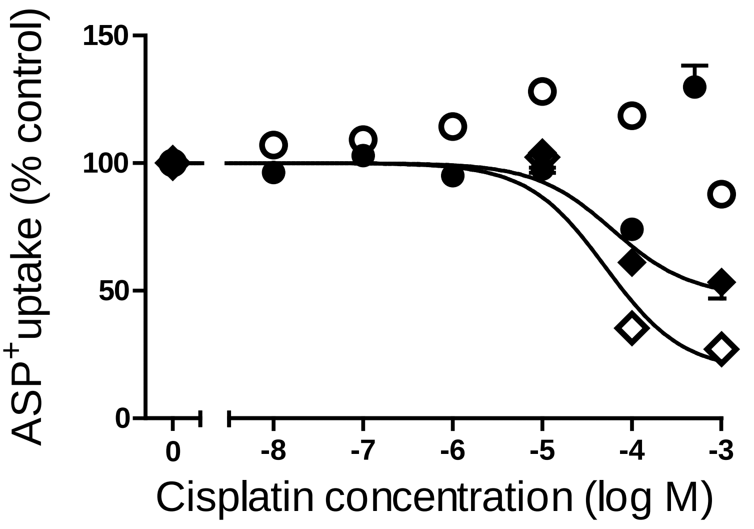

Supplementary materials-Figure 1.

Inhibition of the ASP^+ uptake by cisplatin in HEK293 cells stably transfected with hOCT1 (♦; IC_{50} = 61 µM, n = 21-36), hOCT2 (◊; IC_{50} = 50 µM, n = 18-27), hMATE1 (●; n = 16-40), or hMATE2K (○; n = 16-40).

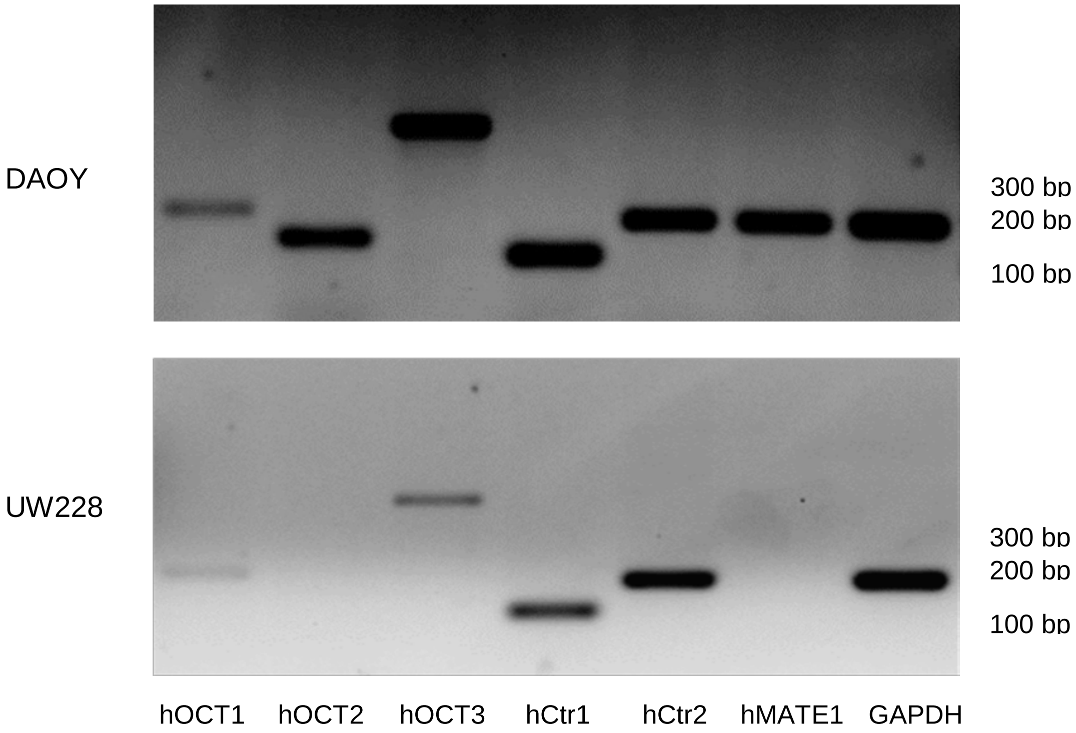

Supplementary materials – Figure 2

Representative PCR analysis of OCT1-3, Ctr1-2, and MATE1 expression in DAOY and UW228 cells. GAPDH as loading control and the base pair (bp) markers are also shown. Compared to DAOY, the UW228 cells do express at low-level hOCT2 and hMATE1.

The sequences (5'-3') of the forward (F) and reverse (R) primers used for the PCR-analysis are:

hOCT1: F- CAT CAT AAT CAT GTG TGT TGG CC; R- CAA ACA AAA TGA GGG GCA AGG CTT

hOCT2: F- CGC CAT TCC TGG TCT ACC GGC; R- GCT TCC TCG ATG GTC TCA GGC

hOCT3: F- GGA ATA GCA TGG TGG AGG ACC A; R- GGG GCT ATG ATT CCC CCA AAA

hCtr1: F- CCT CAC ACT CCC ATG GTG GAG; R- GCT GTA TTG ATC ACC AAA CCG G

hCtr2: F- GGC GAT GCA TTT CAT CTT CTC; R- GGA GGT TGG CAG GTT CAC CAG

hMATE1: F- AAG CTG GAG CTG GAT GCA GTC; R- CAG CAG AGG AGC AGG ACG AGC

GAPDH: F- CAA GCT CAT TTC CTG GTA TGA C; R- GTG TGG TGG GGG ACT GAG TGT GG
